# GenPTM: A Generalizable Framework for Protein Post-Translational Modification Information Extraction from the Scientific Literature

**DOI:** 10.21203/rs.3.rs-9580737/v1

**Published:** 2026-07-08

**Authors:** Shovan Bhowmik, Karen Ross, Chuming Chen, Cathy Wu, K. Vijay-Shanker

**Affiliations:** 1 Department of Computer and Information Sciences, University of Delaware, Newark, Delaware, United States.; 2 Department of Biochemistry and Molecular & Cellular Biology, Georgetown University Medical Center, Washington, D.C., United States.; 3 Center for Bioinformatics and Computational Biology, University of Delaware, Newark, Delaware, United States.

**Keywords:** Post-Translational Modification, Phosphorylation, Text-Mining, BERT, Relation Extraction, BioNLP, Information Extraction

## Abstract

**Background:**

Protein post-translational modification (PTM) plays a pivotal role in cellular activities and biological processes. Although several databases curate PTM information, most cover only a limited number of PTMs. Although the scientific literature continues to accumulate a vast amount of PTM-related knowledge, these databases are not updated regularly. This growing information gap highlights the need for automated information extraction (IE) systems that can identify modified proteins and their specific amino acid sites directly from the literature. While numerous PTMs have been reported in scientific articles, most existing tools are designed only for a few specific PTMs, and developing separate systems for every PTM is not feasible.

**Methods:**

To address this challenge, we developed GenPTM, a generalized and adaptable IE tool that identifies modified proteins and sites from PubMed abstracts using a unified text representation strategy. GenPTM replaces PTM-specific modification and chemical group mentions with generic placeholders, allowing the model to focus on shared textual patterns that express modification events. A BiomedBERT-based classifier is fine-tuned to determine whether a candidate protein or site is truly modified, and a post-processing module assembles the final protein, site, or protein–site pair predictions.

**Results:**

Trained on five major PTM types (e.g., Ubiquitination, Phosphorylation) and evaluated on eight additional PTMs, including PTMs that are not frequently mentioned (e.g., Citrullination, AMPylation), GenPTM achieves F1-scores ranging from 92% to 96% across all PTMs for three different evaluation categories.

**Conclusions:**

These results exhibit strong generalization capability of GenPTM by providing a viable solution for PTM-agnostic IE and automated PTM knowledge discovery in proteomics.

## 1 Introduction

Post-translational modifications (PTMs) are biological events that alter proteins through molecular changes following protein biosynthesis [1]. They represent critical regulatory layers in cellular processes, including signal transduction, transcriptional regulation, degradation, cell-growth and cell metabolism, by profoundly influencing protein stability, localization, interactions, and biological activity [2][3]. For each PTM, there is a special chemical group that attaches to a protein and/or specific sites of a substrate protein, leading to changes in protein dynamics [1][4][5][6]. About 400 various PTMs have been discovered experimentally and a large number of proteins go through these modifications [1][7]. Dysregulation of these PTMs has been linked to diverse pathological states, from cancer to neurodegenerative diseases, making PTM research central to both systems biology and therapeutic discovery [8][9].

Deeper biological insights and many bioinformatic techniques rely on accurate information about which proteins and sites are modified. Information on proteins and sites modified by PTMs is available in several curated databases. However, most recent PTM databases focus on only a limited number of well-documented PTM types. For example, resources such as BioGRID[10] and iPROLink[11] primarily cover a small number of common PTMs, including phosphorylation, acetylation, and ubiquitination. In addition, several PTM-specific databases exist, such as Phospho.ELM[12] for phosphorylation and mUbiSiDa[13] for ubiquitination. Despite their usefulness, existing databases are often not updated. Additionally, information about many PTMs are not found in such databases.

A major limitation of current PTM databases is their heavy reliance on manual curation [14]. With the rapid growth of biomedical literature, it has become increasingly difficult for curators to keep pace with newly published findings, making database maintenance both time-consuming and challenging [15]. Meanwhile, a large amount of PTM-related information continues to be reported in the literature. To systematically extract such information across diverse PTMs and to support timely database updates, automated text-mining approaches for PTM information extraction are highly valuable.

Previous text-mining approaches for extracting PTM-related information from the literature are restricted to specific types of modifications and are not easily generalizable to other distinct PTMs [9][16][17][18][19]. In addition, many PTMs are mentioned only infrequently in the literature, making it difficult to obtain sufficient annotated data to train robust text-mining models for these modifications. Given the biological importance of PTMs, the ongoing discovery of new PTM types, and the limitations of existing resources, there is a clear need for a general-purpose information extraction (IE) framework that can be adapted to automatically extract diverse PTM information (e.g., modified proteins and their sites), directly from the biomedical literature. Such a framework would support database curation efforts and help keep PTM resources up-to-date. In addition, it can facilitate the creation of new PTM-specific resources where no dedicated databases currently exist.

Through a study of PTM mentions in biomedical text, we observed similarity in how PTM-related information is expressed across different modification types. In particular, PTM statements in the literature commonly follow two general forms: (i) the mention of the modification of a protein or site by a specific PTM name, and (ii) the modification of a protein or site through the description of the attachment of a PTM-specific chemical group. Across different PTMs, the underlying sentence structures remain largely similar, with the primary difference in the lexical items used to denote individual modifications.

Motivated by these observations, we developed a general-purpose PTM information extraction tool, GenPTM, that addresses PTM-specific lexical variation through a general placeholder-based representation and extracts PTM information in a PTM-type independent manner. Our GenPTM tool is capable of extracting information for more than 100 distinct PTMs directly from the literature.

Besides, whereas most PTM databases store data from high-throughput experiments, very little information on the context and consequences of those studies is available in these databases. In contrast, GenPTM extracts modified substrates and sites from the literature across diverse contexts as the model is trained on a variety of sentence patterns. Therefore, the data used to develop this model is also valuable for researchers who seek contextual evidence from which PTM-related information is extracted.

In this work, we made the following key contributions:

- We proposed a novel framework to build a PTM IE pipeline that can be adapted to detect any specific type of PTM from the literature.
- We built a large, general PTM corpus corresponding to different PTMs to facilitate model training and evaluation.
- While our framework is designed to generalize across more than 100 PTM types, we evaluated the proposed tool on 13 diverse PTM types, demonstrating the effectiveness and adaptability of our approach in detecting a wide range of PTMs from the literature.
- When applied across different PTM types, our approach achieved the micro-average F1-scores ranging approximately from 92% to 94% over the evaluated PTMs for PTM-modified <site, protein> pair, protein-only and site-only extractions.

## 2 Literature Review

Several databases capture extensive information on PTMs, comprising modified substrates and their specific modification sites. Such databases include UniProtKB [14], CPLM [20], dbPTM [21], and qPTM [22], enabling large-scale studies of protein regulation, signaling pathways, and disease associations. Some databases compiled information about individual modification types. Examples comprise of phosphorylation resources such as Phospho.ELM [12] and PhosphoBase [23]; glycosylation resources such as GlyGen [24], GlycoFly [25]; and ubiquitination resources such as mUbiSiDa [13]. While these repositories are valuable, they suffer from key challenges. Most rely heavily on manual curation of scientific literature, which is time-consuming, resource-intensive, and difficult to scale. As a result, databases lag behind the findings reported in the latest PTM literature, leading to incomplete coverage across diverse PTMs, with information for less-studied PTMs remaining underrepresented.

Researchers have also turned to develop IE systems automatically from unstructured text aiming to extract PTM events. MPTM[26] and EAGL[27] are among the few tools developed for extracting multiple PTM events encompassing phosphorylation, methylation, glycosylation, amidation, etc. While MPTM focuses on identifying PTM-modified proteins and their corresponding sites, EAGL is limited to predicting modification sites. For protein detection, MPTM achieved F1-scores ranging from 47% to 90% and 36% to 93%, respectively. Similarly, EAGL reported F1-scores between 15% and 79% for site prediction. The Turku Event Extraction System (TEES)[17] is one of these tools which modeled PTM IE as a structure event. Although TEES was designed to target a broader set of nine biological events (e.g., gene expression, regulation, binding, and localization) as part of the BioNLP Shared Task [28][29][30], it extracted PTM events, including phosphorylation, ubiquitination, acetylation, glycosylation, hydroxylation, and methylation. While the performance of TEES was the best among all the participants of the BioNLP Shared Task in PTM event extraction, it achieved an F1-score only around 55%. Based on the TEES dataset, both EVEX[31] and BEEDS[32] reframed event extraction as a question answering task, and extracted PTM-specific cause and site. Overall, BEEDS improved recall by more than 14% over EVEX except for Phosphorylation where both tools showed similar recall. BEEDS also improved precision by approximately 4% compared to EVEX. Although it enhanced PTM event recognition, its evaluation was limited to three frequently studied PTMs (Acetylation, Phosphorylation, Ubiquitination).

Building on this, early efforts began to frame the PTM extraction as a Relation Extraction (RE) task where the goal is to link a protein and/or its site with a modification type. Arumugam et al. [33] applied a text mining and machine learning pipeline to detect multiple PTMs, including glycosylation, acetylation, ubiquitination, and hydroxylation. They combined trigger word searches in PubMed abstracts with TF-IDF features and an SVM classifier to capture PTM mentions, substrates, and enzymes, though the lack of reported evaluation limited the impact of their study. Ohta et al. [9] developed a supervised pipeline with three classifiers (trigger, edge, and event detection), presenting a corpus of 400 PTM events covering methylation, acetylation, glycosylation, hydroxylation, and sulfation, though their best F1-score reached only 42%. This study provides a method for extracting mentions of PTMs from text, but its limitations include insufficient data and low performance.

Alongside these general approaches, PTM-specific extraction methods have also emerged. Phosphorylation has been studied extensively, with methods ranging from probabilistic models to hybrid deep learning architectures [34][35][36], and rule-based systems like RLIMS-P [37], specifically mining phosphorylated kinases, substrates and sites from the literature. A deep-learning architecture was proposed to identify ubiquitin E3 ligases and ubiquitinated sites from textual data [38]. However, this study did not report any results or provide details about the dataset used to develop the system. A few text-mining tools were introduced to detect acetylated proteins and sites from texts. A literature mining algorithm was developed to detect acetylation-related patterns for identifying acetylated proteins and sites, achieving 83% precision and 61% recall [39]. However, the results were preliminary and lacked details on the dataset and algorithm. Li et al. proposed a novel lysine acetylation prediction approach using a support vector machine, achieving cross-validation accuracies ranging from 75% to 77% [40]. However, this method was limited to lysine N-acetylation. Although these studies show the effectiveness of the PTM RE task, they are mostly restricted to common PTMs and lack reproducibility.

The advent of transformer-based model adaptations in the biomedical domain, such as BioBERT [41], SciBERT [42], PubMedBERT [43], and BioM-transformers [44], has resulted in state-of-the-art (SOTA) performance on biomedical relation extraction, question answering, and named entity recognition. These models can provide a foundation for PTM detection as a RE task. Early works on protein–protein, drug–drug, and gene–disease interactions utilizing these models demonstrated that supervised approaches can effectively capture structured relations [45][46][47][48]. These advances highlight the strong generalization ability of the transformer models across different relation types. However, to the best of our knowledge, no transformer-based model has been developed for PTM detection.

## 3 Methods

PTM IE plays a critical role in identifying biochemical relationships between proteins, modification sites, and the modifying groups from the scientific literature. Although existing computational tools have primarily concentrated on a limited set of well-studied PTMs (i.e., phosphorylation, ubiquitination, and acetylation), our proposed GenPTM tool is designed to be adaptable to detect any PTM mentioned in text. In this section, we describe the pipeline used to develop the GenPTM tool, illustrate how PTM-specific mentions are generalized across different PTM types and how this framework can be applied for different types of PTM IE.

### 3.1 Approach

PTMs occur when a chemical group is covalently attached to a specific amino acid residue in a protein sequence, known as the substrate, leading to the modification of the protein structure and function [49]. As an initial step, we conducted a study of the PTM literature to understand how modification events are described in the texts. Our investigation of the descriptions of PTM events in the scientific literature reveals that these modification events are typically stated in two main ways:

1. by explicitly mentioning the modification name (e.g., phosphorylation, acetylation)
  - **Example 1:** Acetylation of RXRalpha by p300 facilitated its DNA binding and subsequently increased its transcriptional activity. (PTM: Acetylation) [PMID: 17761950] [50]
    This example indicates a modification (acetylation of the “RXRalpha”) by the explicit use of the modification name ”Acetylation”.
2. by mentioning the corresponding attached chemical group term (e.g., phosphate, acetyl)
  - **Example 2:** Cul-4A is modified by covalent attachment of NEDD8 in rabbit reticulocyte lysates. (PTM: Neddylation) [PMID: 10597293] [51] This example illustrates the second type of PTM description, in which modification (Neddylation) of “Cul-4A” protein is stated through the attachment of the neddylation’s modification group (NEDD8).

Our method is based on the hypothesis that, aside from the modification name or the group name indicating the PTM event, the textual representation describing the modification of a protein or its site remains consistent.

In Example 1 and 2, if these PTM-specific terms are replaced with a generalized placeholder such as “MODIFICATION” or “GROUP”, the resulting sentences can represent modification by any PTM, and the overall PTM statement becomes identical regardless of the specific modification type. To illustrate this process, consider the generalized representations for both examples:

#### Generalized Representation (Example 1):

MODIFICATION of [E]PROTEIN[/E] by p300 facilitated its DNA binding and subsequently increased its transcriptional activity.

#### Generalized Representation (Example 2):

[E]PROTEIN[/E] is modified by covalent attachment of GROUP in rabbit reticulocyte lysates.

From the original sentence (Example 1), we create an instance in which the PTM-specific mention “Acetylation” is replaced with the generic placeholder “MODIFICATION”, and the protein mention “RXRalpha” is replaced with a feature term (f-term). The f-term is a biologically meaningful descriptor that represents the functional class of the protein and is explicitly marked as [E]PROTEIN[/E] [52]. This representation strategy allows the model to focus on learning relational patterns rather than memorizing entity-specific lexical cues. The rationale behind this generalized representation is discussed in more detail in Section 3.2.1. Likewise, we can also create an instance for Example 2 by replacing “NEDD8” with “GROUP”.

As an illustration, if we consider another PTM type, “Phosphorylation,” in Example 3, the modification event follows a similar textual pattern like Example 1, except that it mentions a different PTM type. where GAIP is phosphorylated and the event is expressed using the modification name itself.

**Example 3:** Phosphoamino acid analysis revealed that phosphorylation of GAIP occurred largely on serine residues. (PTM: Phosphorylation) [PMID: 10760275] [53]

In both cases (Example 1 and Example 3), the sentence structure describing the modification event remains the same; only the specific modification term changes. Therefore, the generalized representation for Example 3 will have a similar textual pattern:

#### Generalized Representation (Example 3):

Phosphoamino acid analysis revealed that MODIFICATION of [E]PROTEIN[/E] occurred largely on serine residues.

The examples stated above justify our hypothesis that modification events can be expressed in a PTM-agnostic manner by replacing either the modification name or the corresponding chemical group terms with generic placeholders. Training a language model on such generalized representations allows the system to capture patterns that enable the extraction of modification relations across diverse PTM types.

We manually reviewed 100 abstracts spanning 10 different PTMs to confirm how frequently these two forms of mention occur. Nearly every sentence that referred to a PTM event fell into one of these types. Notably, in 99 out of the 100 abstracts, whenever a modification of a site or protein was mentioned, it was stated at least once using one of these two forms.

While this generalization strategy applies to most PTMs, we exclude a few special cases (e.g., methylation, where the modification-related terms can refer to both protein and DNA methylation and glycosylation, where diverse structure terms can act as the attached group; and reverse modifications that remove a group rather than attach one), which we will discuss further in Section 6.

### 3.2 Training Pipeline

**Fig. 1:**
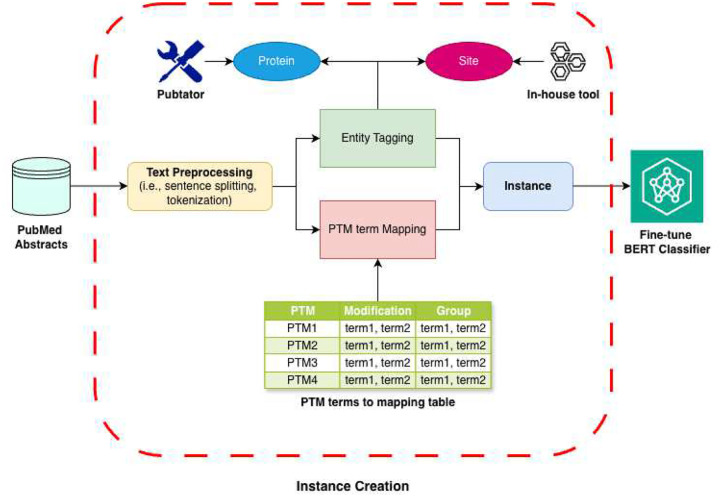
Overview of the GenPTM Training Pipeline. In the training pipeline, PubMed abstracts are first preprocessed through sentence splitting and tokenization. Proteins are annotated using PubTator, while sites are detected using an in-house site recognition tool. PTM mentions are replaced with a generic placeholder using a PTM-term mapping table. Candidate entity mentions are then marked within sentences to generate instances. These instances are used to fine-tune a BERT-based classifier to learn whether a marked entity represents a true modified protein or a modified site.

The core component of our training pipeline is instance creation, where each input sentence is converted into a PTM-agnostic, generalized representation that can be used to learn modification relations across PTM types. Fig. 1 illustrates the overall training pipeline of our GenPTM tool. In the following subsections, we describe the details of instance creation and the fine-tuning of the language model used to learn these generalized PTM statements.

#### 3.2.1 Instance Creation

As input, we use PubMed [54] abstracts related to PTMs, retrieved using a predefined set of PubMed identifiers (PMIDs). The corresponding abstracts are downloaded via the Entrez API^1^ and subsequently processed through a text preprocessing stage. This stage includes sentence segmentation and tokenization using spaCy^2^, enabling sentence-level prediction of protein or site modification. Each sentence is then independently considered for downstream processing.

Following text preprocessing, named entity recognition (NER) is performed to identify candidate proteins and modification sites within each sentence. Protein entities are annotated using PubTator3 [55], which provides SOTA biomedical entity recognition. To detect site mentions, we develop an in-house tool that leverages a set of carefully designed regular expressions to capture different amino acid patterns (e.g., serine-763, Lys350, serine residue at 209) commonly found in the literature.

Once the protein and site entities are recognized, the next step is to identify the PTM mentions and normalize them into generalized modification terms to achieve a unified textual representation across different PTMs. The normalization of PTM-specific modification names and chemical group mentions using a mapping table is described below.

##### PTM Mapping

As discussed earlier, PTM events are typically expressed through modification names (e.g., phosphorylated, acetylation) or group names (e.g., ubiquitin, SUMO), each indicating the type of modification in the sentence. To make the system adaptable for any PTM type, these mentions are replaced with generalized placeholders, “MODIFICATION” for modification names and “GROUP” for chemical groups or moieties, using a curated mapping table of PTM-specific keywords and their corresponding generic forms. Table 1 highlights the mapping of PTM-specific mentions for 13 PTMs used in the evaluation of this work (See Section 4.3) in the literature that could be replaced with a generic mention.

We also provided a comprehensive mapping table containing more than 100 PTMs for which our tool can be adapted to extract information for these PTMs and from this table we selected 13 representative PTMs for this study (Table 1). The complete table is available in supplementary section S1.

Although the table lists only the “MODIFICATION” class, we adapt the generalized representation based on the part-of-speech of the original PTM mention to preserve grammatical correctness. For instance, in a passive construction such as “X can be phosphorylated”, the term “phosphorylated” is replaced with “MODIFIED”, whereas in an active form like “Y phosphorylates X”, it becomes “Y MODIFIES X”. However, we always use “GROUP” to substitute any PTM-specific group mention.

By performing this PTM mapping, the text is standardized in such a way that the language model can focus on the relational structure between modification cues and entities. This step is crucial for enabling the adaptive PTM information extraction framework to recognize diverse modification types using a general representation.

##### Model-Ready Instances

Once the entities are tagged and the PTM-specific mentions are normalized, each sentence is transformed into a modified sentence representing a generalized textual pattern. These modified sentences are instances that serve as the standardized inputs for the training model, enabling it to learn the underlying relational structure between the marked entity and the modification cue. In each instance, only one entity is marked at a time, even if multiple entities are present, so that the model predicts the relation specifically between the marked entity and the modification term. During training and inference, the model uses these instances to predict whether the sentence expresses a positive or a negative modification relation.

While creating the instances, each candidate protein is replaced by an f-term, PROTEIN and each candidate site by SITE, to indicate the focus of classification by the language model. We normalize specific protein mentions to a generic term (PROTEIN) to prevent the model from learning biases toward frequently occurring proteins in the training data. Since the same protein can appear multiple times across the corpus, this normalization discourages memorization of protein-specific lexical cues and encourages the model to focus on learning general modification relations. Similarly, we normalize the specific site mentions to a generic term (SITE) to avoid the lexical bias toward individual amino acids. This action reduces data sparsity and enable the model to focus on the semantic relationship between the modification and/or group name and the candidate entity rather than focusing on specific protein or site mentions. Besides, each candidate entity (PROTEIN or SITE) is further enclosed within special boundary markers [E] and [/E], which explicitly highlight the target of classification and guide the model’s attention to the entity under consideration.

**Fig. 2:**
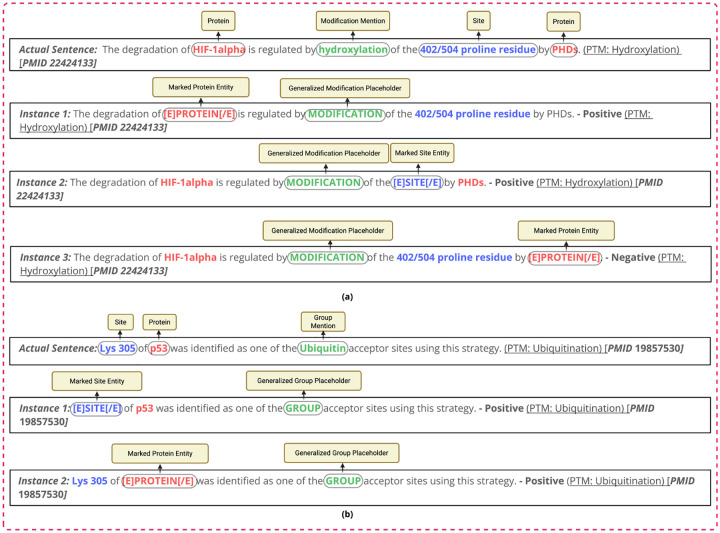
Input representation for PTM–substrate relation extraction. (a) Hydroxylation of HIF-1alpha at two proline residues is generalized by replacing the modification mention (hydroxylation) with the placeholder MODIFICATION and generating multiple candidate-specific instances. In Instance 1, the protein (HIF-1alpha) is marked as [E]PROTEIN[/E], resulting in a positive example. In Instance 2, the site (402/504 proline residue) is marked as [E]SITE[/E], also forming a positive instance. In Instance 3, an unrelated protein mention (PHDs) is marked as [E]PROTEIN[/E], resulting in a negative instance, as it does not represent the modified substrate. (b) (b) Example sentence describing ubiquitination of p53 at Lys 305 with the group mention is generalized as follows: the group term (Ubiquitin) is normalized to GROUP, and the candidate entities are modified in two instances, where the site mention (Lys 305) is replaced as [E]SITE[/E] in Instance 1, and the protein substrate (p53) is replaced as [E]PROTEIN[/E] in Instance 2, both resulting in positive instances. In both cases, entity replacement and PTM mapping transform diverse PTM mentions into a unified format, allowing the model to learn shared textual patterns across PTMs. The resulting modified sentences, containing both generalized PTM mentions and marked entities, are used as inputs for classification.

During training, to avoid bias only towards modification events, each instance is labeled as positive or negative based on the presence of a modification relation between the modification (or group) term and the candidate entities within the sentence. An instance is considered positive when the modification term is explicitly associated with a protein or site mention. In contrast, if the sentence does not indicate any specific protein or site being modified, the instance is labeled as negative. Fig. 2 shows two representative examples of model-ready instances that we created.

We want to emphasize here that we create instances only from sentences that contain both a marked entity (protein or site) and a modification-related term (modification name or chemical group), and we ignore any sentence missing either element for instance creation. In addition, the pipeline relies on PubTator for protein recognition, and consequently, missed protein annotations (if any) by PubTator can lead to missed PTM IE.

#### 3.2.2 Transformer-based Language Model

**Fig. 3:**
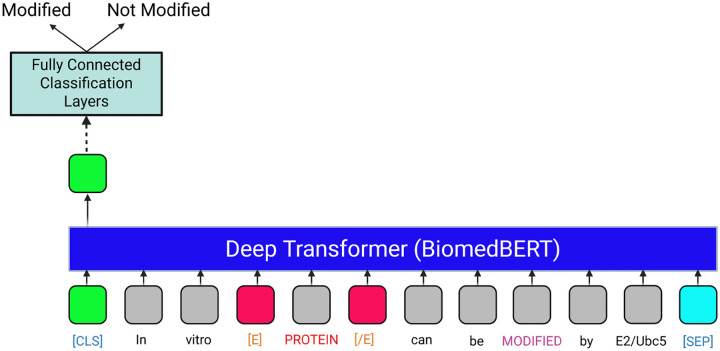
Overview of the BERT-based Training Model. This language model utilizes the embedding of the [CLS] token as the sentence-level representation, which passes through fully connected classification layers to predict whether a given entity is modified.

For the training and prediction of our GenPTM tool, we fine-tuned a transformer-based language model, BiomedBERT [56], which has been pre-trained on large biomedical corpora and is well-suited for domain-specific relation extraction (RE) tasks. Although there are some other domain-specific BERT architectures, e.g., BioBERT [41], SciBERT [42], we prefer to use BiomedBERT as this is pre-trained on PubMed articles and hence, more suitable for this task. The PTM entity prediction task is formulated as a binary classification problem, where the model determines whether a marked entity (protein or site) is modified in a given sentence. Our BERT-base transformer model is illustrated in Fig. 3 and described below.

We fine-tune the BiomedBERT base model for this task by extracting the embedding of the special [CLS] token from the final hidden layer (a 768-dimensional vector), which represents the entire input sentence. This embedding is passed through a fully connected linear layer of size 768×512, and then through a classification layer of size 512×2. A softmax activation function produces the probability distribution over the two classes (“modified” and “not modified”).

We optimized our model by using the Adam optimizer with a learning rate of 2*e* − 5, L2 regularization of 0.001, and trained for 10 epochs with a batch size of 16. The cross-entropy loss function is used as the objective, and an 80/20 train-validation split ensures stable hyperparameter tuning based on validation loss and accuracy.

We employed the pre-trained BiomedBERT weights available on HuggingFace^3^ as the base model for PTM modified protein and site prediction. All experiments were executed on a workstation equipped with an NVIDIA A100 PCIe GPU (40 GB memory) and 64 GB of system RAM, providing adequate computational resources for large-scale fine-tuning. This setup ensured stable training, faster convergence, and reliable model performance across diverse biomedical abstracts. This trained model is the backbone of our GenPTM tool.

### 3.3 Inference Pipeline

Once the BERT-based language model is trained, we use this fine-tuned model on unseen abstracts for any target PTM. For a chosen PTM, we follow the same “Instance Creation” step described in Section 3.2.1 to create model-ready instances. Each instance is then passed to the fine-tuned model to decide whether the marked entity is modified. Instances predicted as positive are further post-processed to finally obtain the protein and site modified by any specific PTM.

This step is necessary because the language model works at the instance-level with a single marked entity, which could be a representation of an f-term, or only a mention of a site of a protein. Therefore, post-processing has two key goals: (i) resolve the explicit substrate or site mention if the model prediction is made on a generalized f-term, and (ii) for a given abstract, correctly associate modified sites with their corresponding substrate proteins when a site mention is predicted. This ensures that all extracted entities ultimately contain the precise modified protein and/or the exact modification site.

**Fig. 4:**
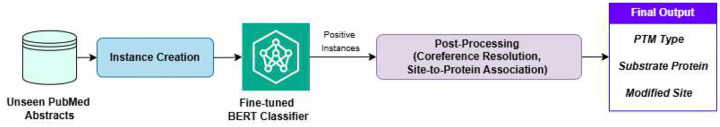
Overview of the GenPTM Inference Pipeline. In the inference pipeline, unseen PubMed abstracts are processed following the same “Instance Creation” module to ensure consistency between training and inference. The generated instances are passed to the fine-tuned BERT classifier to identify positive protein or site predictions. A post-processing step subsequently resolves coreference and associates modification sites with their corresponding proteins. The final structured output reports the PTM type, the modified protein, and the specific modification site.

Our inference pipeline is shown in Fig. 4. The post-processing steps (Coreference Resolution and Site-to-Protein Association) are described in the below subsections.

#### 3.3.1 Coreference Resolution

In some biomedical literature, PTM substrates are not always directly mentioned within the same sentence. Instead, they may be expressed through pronouns, or referential phrases such as “this protein”, “its”, “these residues”, etc. To address this, we perform coreference resolution to map these referring expressions back to the correct protein or site in the surrounding context (current or prior sentences of the abstract). We illustrate this with the following example:

**Example 3:** A complex signalling cascade involving GSK3beta kinase, the Pin1 prolyl isomerase, and the PP2A-B56alpha phosphatase controls phosphorylation at these sites. [PMID: 19131971] [57]

In Example 3, “these sites” being phosphorylated are not stated in this sentence. Instead, they appear in the previous sentence of the abstract: “In cancer cells, c-Myc can become aberrantly stabilized associated with altered T58 and S62 phosphorylation.” Thus, the reference for “these sites” (the residues “T58” and “S62”) is retrieved from earlier context. We resolve this by first looking for an explicit protein or site in the same sentence, then in the immediately preceding sentence, and, if necessary, in the title or opening sentence of the abstract, ensuring that each predicted PTM relation is linked to a concrete biological entity.

#### 3.3.2 Site-to-Protein Association

Since our trained model is designed to predict one entity at a time, if the predicted entity corresponds to a site, without determining the substrate protein it belongs to, the prediction is of no use. This post-processing step enables us to convert site-level predictions into complete protein–site modification pairs, which are required for an accurate PTM information extraction framework.

**Example 4:** Mechanistic studies revealed that CSN6 is deregulated by epidermal growth factor receptor (EGFR) signaling, in which ERK2 binds directly to CSN6 Leu163/Val165 and phosphorylates CSN6 at Ser148. [PMID: 26267535] [58]

For instance, Example 4 has two entities, the site “Ser148” (colored in blue) and the protein “CSN6” (colored in red). As our model can predict only the modified site or the protein at a time, this post-processing step allows us to establish the associated substrate for the site so that the final output of the pipeline includes not only the modified site (e.g., Ser148) but also the corresponding protein it belongs to (e.g., CSN6).

From our investigation, we observed that, in many cases, the residue and its substrate are mentioned within the same sentence and they follow similar patterns in the text. For such instances, we analyze the dependency and part-of-speech (POS) tags of the sentence using “spaCy” and develop a series of high-precision grammar-based rules to associate sites with their corresponding proteins. These rules cover a wide range of syntactic structures, including active, passive, and nominalized forms of sentences and other linguistically stable patterns that frequently occur in biomedical literature. We follow the rules used in the Extended Dependency Graph (EDG) tool [59] to design these precise rules.

Additionally, we also handle generic site mentions, propagating associations across conjunctively listed sites or proteins, resolving cases involving intermediate regions or domains, and extending associations across sentences. Together, these steps ensure that site-level predictions are consistently converted into correct protein–site modification pairs. Detailed descriptions of these rules and patterns are provided in supplementary section S2.

## 4 Experimental Setup

This section presents the construction of the corpus used for developing the BERT-based Generalized PTM IE Framework, along with the statistics of the training and testing datasets.

### 4.1 Corpus Construction

For developing the GenPTM framework, it is essential to create a comprehensive corpus for model training and evaluation. To assess the adaptability of the model on diverse PTM types, annotated datasets are prepared for differt PTM types. Three expert annotators collaboratively worked on the selected abstracts following a structured annotation protocol. The annotation process was PTM-specific, meaning that for a given PTM type *P*, the annotators considered only events corresponding to that PTM *P*, even if other PTMs appeared in the same text. If a sentence contained more than one entity (protein or site), the annotators annotated each instance separately, treating each entity in the sentence as an independent annotation instance. For example, if a sentence consists of four entities, four decisions were made separately, each focusing on a single entity. Disagreements between annotators were resolved through discussion to reach a final decision. In cases where a final decision was not achieved, the instance was reviewed by another expert with extensive PTM research experience, who provided the final decision.

### 4.2 Training Dataset

To construct the training corpus for developing the classification model, we compiled annotations from five well-studied distinct PTMs: Ubiquitination, Ubiquitin-like PTMs (Sumoylation and Neddylation), Acetylation, and Phosphorylation. We selected these PTMs to examine whether these widely reported modifications are broad enough to capture PTM-related IE from the literature in general. Therefore, we used abstracts for these PTMs only when constructing the training dataset. Abstracts for these PTMs were collected from established repositories, iProLINK [11] and BioGRID [10]. In addition, because of the textual diversity observed in ubiquitination-related literature, we expanded the training dataset of Ubiquitination by retrieving additional abstracts from PubMed using the query “Ubiquitinat* OR Ubiquitylat*”. These categories are selected to ensure coverage of diverse textual and contextual representations of PTM events. In total, 395 abstracts were considered, yielding 4043 annotated instances including 1720 positive instances and 2323 negative instances.

### 4.3 Testing Dataset

To evaluate the model’s ability to generalize across PTM types, we constructed a diverse testing corpus containing 13 PTMs organized into three groups. The first group includes well-studied PTMs that we used to construct the training corpus. We refer to this set of five PTMs as Well-Studied PTMs (WS-PTMs). To examine generalization to unseen modifications, we further considered two additional groups that were not included in the training data. The second group, refered to Other Frequent PTMs (OF-PTMs) and consists of other frequent PTMs well-reported in the literature, including Hydroxylation, Succinylation, Palmitoylation, ADP-Ribosylation, and UFMylation. The third group contains three less-reported PTMs (AMPylation, Citrullination, and Lipoylation) in the literature and named as Low-frequent PTMs (LF-PTMs). For the WS-PTMs, abstracts were collected from the same databases used to obtain the primary training corpus. For the OF-PTMs, abstracts were retrieved directly from PubMed using query structures described in Section 4.2, incorporating PTM-specific lexical prefixes. We took a random subset of abstracts from PubMed for annotation. However, certain LF-PTMs were underrepresented in the literature compared to OF-PTMs. Besides, the available abstracts did not provide sufficient protein and/or site-level information. Consequently, we retrieved a combined total of 68 abstracts across these LF-PTMs from the dbPTM database [21].

Based on the availability of protein and site mentions, we selected the number of abstracts for the PTMs used in our experiment. The final testing corpus consists of 488 abstracts, serving as the basis for assessing the model’s generalization and cross-PTM adaptability. The number of abstracts available per PTM type varied, reflecting differences in literature availability that explicitly mention both the PTM and its modified entity. Nonetheless, for each PTM type, we aimed to maintain a balanced distribution between positive and negative instances. Altogether, the testing corpus contains 417 unique truly modified proteins and 183 unique truly modified site annotations. We excluded other protein and site mentions present in the abstracts from this count. Nevertheless, those mentions were part of the evaluation process. Moreover, because some entities are mentioned multiple times within abstracts, the total number of instances is considerably higher, reaching 3142 instances, of which 1465 are positive and 1677 are negative. The statistics of the testing dataset across different PTMs are summarized in supplementary section S3 (See Table S2).

### 4.4 Evaluation Strategy

Although our language model predicts PTM-modified sites or proteins at the instance level, this does not represent the final output of the GenPTM system. The end-to-end GenPTM pipeline operates at the abstract level, extracting modified proteins, sites, and their associations from the entire abstract. Therefore, we evaluated GenPTM at the abstract level to determine whether it successfully extracts PTM-modified proteins, sites, or protein-site pairs.

We considered three evaluation criteria:

- <**protein, site**> **pair:** This criterion evaluates whether our tool correctly predicts both a modified site and its associated protein within an abstract. If a modified site is present, we assume that the associated protein is also mentioned in the same abstract, either in the same sentence or elsewhere. A prediction is considered correct only when the system accurately detects the site and correctly links it to its corresponding protein.
- **protein-only:** This criterion evaluates whether the system correctly predicts a PTM-modified protein mentioned in an abstract, regardless of whether the abstract also reports a specific modified site for that protein. Under this criterion, a prediction is considered correct if the system successfully extracts the modified protein from the text. This evaluation allows us to assess how well the tool detects modified proteins in cases where only protein-level modification information is required without necessarily specifying their modification sites.
- **site-only:** This criterion evaluates whether the system correctly identifies a PTM-modified site mentioned in an abstract, regardless of whether the associated protein is explicitly detected by the system. Similar to the protein-only criterion, a prediction is considered correct if the modified site is accurately extracted from the text. This evaluation allows us to assess the model’s ability to detect modification sites independent of the substrates.

Note that the last two evaluation criteria focus solely on assessing the ability of the BERT model and our underlying hypothesis to detect modification mentions. In contrast, the <protein, site> pair extraction task further incorporates our heuristic-based method to evaluate whether the system can correctly associate a mentioned site with its corresponding substrate.

For each of the three evaluation criteria, we compute True Positives (TP), False Positives (FP), and False Negatives (FN) at the abstract level.

Since this is an abstract-level evaluation, we count each protein or site at most once per abstract, even if it is mentioned multiple times. Therefore, if a modified entity is extracted from any single mention in the abstract, it is considered a successful extraction for that entity. Similarly, we count each ⟨protein, site⟩ pair at most once per abstract, even if the relation is obtained from multiple mentions. This criterion reduces the total number of counted entities because only unique entities are considered. Consequently, if an abstract contains only one mention of a protein or site and it is missed or extracted incorrectly, it can disproportionately affect the overall performance.

## 5 Results

We adopt abstract-level evaluation because GenPTM is designed as an end-to-end system where the input is a PubMed abstract and the output is the collection of extracted modified proteins and sites. In this approach, the key objective is whether the system can successfully identify the modified entities in the abstract, regardless of how many times they are repeated in the text. Since the intended use of the system is to build PTM information database by extracting modified proteins and sites by different PTM types, abstract-level evaluation provides a realistic measure of its effectiveness for large-scale literature mining. Additionally, some of our test datasets were derived from the dbPTM database [21], which provides PTM information only at the abstract level. In such cases, only the modified entities associated with an abstract are available rather than sentence-level annotations. Therefore, abstract-level evaluation is appropriate for these datasets and also aligns well with the design of existing PTM databases (e.g., UniProtKB [14], dbPTM [21], and qPTM [22]), which typically record the modified entities, the PTM type, and the corresponding PMID without preserving individual mentions from the literature. Furthermore, we also report instance-level evaluation across diverse PTM-categories, along with PTM-wise results in supplementary section S5 (See Table S5 and Table S6), in order to assess the performance of the fine-tuned BERT model in the GenPTM framework.

To evaluate performance across different PTM categories, we report micro-averaged Precision (P), Recall (R), and F1-score (F1), which are standard metrics in machine learning [60]. Results are presented for three PTM categories: (i) WS-PTMs, (ii) OF-PTMs, and (iii) LF-PTMs, as well as for all PTMs (Table 2). PTM-wise results for each evaluation criterion are provided in supplementary section S4 (See Table S3).

From Table 2, we can observe that GenPTM achieves strong abstract-level performance across all PTM categories. Performance is stable not only for the WS-PTMs but also for the OF-PTMs, indicating good generalization beyond the PTMs seen during training. However, LF-PTMs show a noticeable drop. In the next section, we analyze these trends, highlight common sources of errors, and discuss the constraints of the current pipeline.

## 6 Discussion

### 6.1 Result Analysis

The experimental evaluation shows that GenPTM generalizes effectively across diverse PTM types, achieving F1-scores above 92% under all three evaluation criteria. Although the model was trained on only five PTM types, it successfully extended to the OF-PTMs and LF-PTMs categories, thereby supporting our central hypothesis. If we notice individual PTMs (see Table S4), we find that, with a few exceptions, the precision and recall for most of the PTMs in our test dataset are at or close to 90%. Additionally, for most WS-PTMs and OF-PTMs, the model successfully extracted modified <protein, site> pairs and sites with near 100% precision and 90% recall.

For OF-PTMs, the system achieved an F1-score exceeding 91%, with 100% site precision across the evaluated abstracts. Besides, the <protein, site> pair evaluation showed strong and consistent performance. In contrast, while the protein-only precision and recall for OF-PTMs are high, the precision is slightly lower compared to the WS-PTMs. On closer examination, we observed that the lower performance for OF-PTMs in the protein-only evaluation was primarily attributed to the results for UFMylation and ADP-Ribosylation. In the case of ADP-ribosylation, although the number of abstracts in the test corpus was comparable to other OF-PTMs, the number of truly modified proteins was limited as some abstracts did not contain any ADP-Ribosylated proteins. As a result, a few incorrect predictions led to a noticeable drop in precision. On the other hand, 60% of the precision errors for UFMylation were stemmed from a single sentence within an abstract that contained multiple protein mentions [61]. In that sentence, multiple proteins were mentioned together as components of the UFMylation pathway rather than as substrates undergoing modification. The sentence is as follows: “Total RNA and proteins were extracted from tissues to examine the expressed patterns of UFMylation components, including UBA5, UFC1, UFL1, DDRGK1, UFSP1, UFSP2 and UFM1, by real-time PCR and western blot analysis”. All seven unique proteins in this sentence were not annotated as modified entities for this abstract; however, the system incorrectly predicted them as modified proteins, leading to a noticeable drop in precision. Apart from these 7 FPs, the remaining 5 FPs originated from the other 39 abstracts.

While performance for WS-PTMs and OF-PTMs is robust across all evaluation criteria, LF-PTMs show comparatively moderate performance, with F1-scores still approaching 90% for protein-only and site-only evaluations. For this category, we observed reduced precision and recall for <protein, site> pair evaluation and decreased precision for site-only evaluation (See Table 2). Again, this decline is mainly due to the limited number of protein and site mentions available for these PTM types. As a result, just a few sentences with complex structures and the enumeration of multiple entities contributed to all the errors for this PTM category.

Among all these errors for LF-PTMs, we will first address the decreased precision of <protein, site> pair extraction. For example, in the case of Citrullination (also known as Deimination), one abstract [62] containing the following sentence describes multiple modifications across different protein components: “Other modifications found in bovine MBP include N-terminal acetylation in components C1, C2, and C3; oxidation of methionine 19 in all five components; all charge isomers having both a mono- and dimethylated (symmetric) arginine at position 106; deimination in arginines 23 and 47 found only in component C8b; deimination of arginine 96 and deamidation in glutamine 102 found in components C2, C3, C8a, and C8b; phosphorylation in threonine 97 restricted to charge components C2 and C3; deimination in arginine 161 only found in component C3; deamidation of glutamine 120 was only observed in C3.”

The drop in precision for the <protein, site> pair evaluation was due to eight FP pairs originating from a single sentence. Two distinct factors contributed to these errors. First, the complex sentence structure involving multiple PTMs confused the system to incorrectly predict methionine 19, arginine 106, glutamine 102, and threonine 97 as citrullinated sites although simpler sentences with fewer PTM mentions (e.g., acetylation and phosphorylation within the same sentence) did not lead to such errors. Second, the remaining four FPs were not due to prediction errors, but rather arose from limitations of the NER tool used for protein detection. Specifically, the predicted sites were incorrectly associated with MBP, which refers to a protein complex, rather than the individual protein components (e.g., C2, C3, C8a, and C8b) to which these sites actually belong.

The same issue (detection of protein components) caused the drop in recall for the <protein, site> pair evaluation in Citrullination. For instance, in [63], the sentence “Component C3 contains eight phosphorylated sites (S7, S33, S64, T96, S113, S141, S164, and S168), and citrulline residues at Arginine 41, R24 and R165,” the citrullinated sites (e.g., arginine 41, 24 and 165) were correctly extracted by the system. However, the NER tool failed to recognize the corresponding protein component (C3), which consequently prevented the assignment of the modified sites to the correct protein, leading to missed <protein, site> associations. A similar situation was also observed in another sentence in the same abstract. Together, these two sentences account for approximately 50% of the recall errors for Citrullination within the LF-PTMs category.

### 6.2 Comparison with the State-of-the-Art Tools

Although benchmarking against existing baselines is desirable, we did not identify a publicly available, standardized text-mining dataset that would allow a proper comparison with existing tools. Most of them were evaluated on non-public datasets. In many cases, the evaluation corpora are not downloadable or the original evaluation platforms are no longer operational. As a result, we could only perform approximate comparisons using reported results from the literature.

As discussed in Section 2, MPTM [26] and EAGL [27] are among a few tools that incorporated multiple PTMs in their evaluation. MPTM was evaluated on reannotated abstracts from the iProLink database [11] (publicly unavailable), reported F1-scores of 90% (protein) and 93% (site) for phosphorylation, and 80% and 91% for acetylation. EAGL, focusing only on site extraction, achieved lower performance (72% for phosphorylation and 55% for acetylation). Since MPTM used iProLink abstracts containing Phosphorylation and Acetylation information and we independently collected abstracts for the same PTMs from the same database, we conducted an approximate comparison. Using the same collection of abstracts but with additional PTM-specific annotations, GenPTM showed notable improvements about +7% (protein) and +5% (site) over MPTM for phosphorylation, and +12% and +1% for acetylation, with much larger improvements over EAGL. Unlike these systems, our framework also evaluates <protein, site> pair extraction, which is not evaluated for these systems.

Additionally, the BioNLP Shared Task 2011^4^ [29] was a major community effort where one of the tasks focused specifically on PTM event extraction, where systems were required to identify the Theme (modified protein), and the corresponding Site across multiple PTM types. Several systems were evaluated in this challenge, including the Turku Event Extraction System (TEES) [17], EVEX [31], BEEDS [32] and EventMine [64], which reported performance in 50%−70% range on PTM event extraction. Again, we were unable to access the annotated evaluation set, and the official evaluation platform was no longer available, preventing us from performing a direct experimental comparison. Although a strict comparison is not feasible, the example sentences provided in the shared task closely resemble the textual patterns present in our training and testing corpora. While the highest reported F1-score for PTM event extraction in that task was 53.33%, GenPTM achieved an F1-score of 91.30% for the <protein, site> pair across multiple PTM types on our dataset.

Similarly, the BioNLP Shared Task 2013[30] was another effort that introduced the Genia Event Extraction (GE) task which included phosphorylation event extraction. However, this dataset is also no longer available on the official website^5^. Nevertheless, we found that RLIMS-P 2.0[65] systematically outperformed other SOTA tools on this GE dataset on extracting phosphorylated substrates and their corresponding sites from biomedical literature. Because RLIMS-P was developed in our laboratory and its evaluation dataset is accessible, we were able to perform a direct comparison for Phosphorylation. GenPTM improves over RLIMS-P with +2% precision and about +7% recall (reaching 96%). We also tried to examine PTM-specific extraction tools developed for Acetylation [33] and Ubiquitination [38]. However, these systems did not provide annotated corpora or detailed evaluation results.

Finally, it is important to note that GenPTM was trained on a newly constructed annotated corpus, uses a unique input representation based on generalized placeholders, and is evaluated across a heterogeneous set of PTM types. In addition, our evaluation includes PTMs that have not been addressed by previous text-mining systems, such as UFMylation, AMPylation, Citrullination and Lipoylation. Due to these differences in datasets, evaluation protocols, and PTM coverage, the comparisons presented here should be interpreted as indicative rather than strictly equivalent benchmarks.

### 6.3 Application Constraints

Although our generalization framework is effective for a wide range of PTMs, several modification types were excluded from training and evaluation due to a few additional challenges.

First, Glycosylation was excluded as our current approach relies on a fixed set of modification group names associated with each PTM type. In contrast, glycosylation is expressed through highly diverse glycan structural terms rather than a single standardized group name. For example, glycosylation can be described using structural terms such as “triantennary structures”, “O-linked oligosaccharides”, or “sialyl Lewis X” [66][67], which function as the modification GROUP terms considered in our approach. However, unlike other PTMs where a small fixed set of GROUP terms is sufficient, glycosylation involves a vast and highly diverse set of glycan structure terms that can act as modification groups. Therefore, accurately capturing such expressions would require a dedicated Named Entity Recognition (NER) system capable of detecting the wide range of glycan structure terms that act as modification groups.

Moreover, glycosylated substrates or sites are often expressed through indirect verbal cues rather than explicit modification names. For example, modified proteins or sites may be described using words such as “occupied” or “utilized,” where glycosylation is implied but not directly mentioned. We did not treat such expressions as modification names for glycosylation in our current system. To address this limitation, the framework would need to be extended by combining transfer learning from the current model with additional learning from glycosylation-specific corpora to train a dedicated system for the glycosylation IE. Another challenge is the frequent use of terms such as “potential,” “putative,” or “consensus,” which typically indicate predicted glycosylation sites rather than confirmed modification events. Since these mentions do not necessarily correspond to true modified entities and not usually observed in other PTMs, glycosylation requires separate treatment.

Besides, another PTM, Methylation was excluded from evaluation. The primary challenge arises from ambiguity between protein methylation and DNA methylation, as the same lexical trigger “methylation” can refer to distinct biological processes. Disambiguating these events requires additional processing beyond the current scope of our system.

Finally, we did not consider PTM removal events, often referred to as demodifications, such as dephosphorylation, deubiquitination, and deacetylation. These processes reverse the corresponding modifications by removing the attached group from the substrate or site. Because GenPTM is designed to detect modification events involving the addition of groups to substrates or sites, the current framework does not address these demodification events.

Overall, while GenPTM demonstrates strong generalization across diverse attachment based PTMs, these excluded categories highlight specific challenges that need dedicated effort in future extensions of the system.

## 7 Conclusion

In this work, we developed a general-purpose PTM information extraction framework capable of identifying PTM-modified substrates and sites from the biomedical literature. To achieve this, we constructed a large, generalized corpus incorporating diverse and well-known PTMs expressed through varied textual patterns, enabling the model to generalize effectively to unseen PTM types. We evaluated the framework not only on PTMs included during training but also on several additional and very less-frequent PTMs. The results show strong generalization capability, achieving F1-scores exceeding 92% for protein, site, and <protein, site> pair extraction.

Unlike most existing tools, which are usually built for specific PTM types, our framework is designed to handle a wide range of PTMs in a single system. It performs uniformly well across different PTM categories, achieving F1-scores above 90% in most cases, while existing approaches on similar tasks typically report results in the 70–80% range. For LF-PTMs, the performance is slightly lower (around 85%) mainly due to limitations in the NER component used to detect entities, rather than the prediction model itself.

Our study specifically focused on how PTM information is expressed in scientific text. We validated our central hypothesis that replacing PTM-specific modification and group mentions with generic placeholders is sufficient to learn textual patterns that can recognize modified proteins and/or sites. To our knowledge, no other tool currently leverages textual abstraction to develop a PTM-agnostic IE framework.

Although our evaluation covered 13 PTMs, the mapping table suggests that the framework can be extended to many additional PTMs, provided their modification-specific mentions can be standardized into generalized placeholders. However, as discussed in Section 6, the current framework does not yet support PTMs such as Glycosylation and Methylation due to their structural and contextual complexities. Future work will focus on incorporating these PTMs and extending the model to handle reverse modification events.

Given the critical role of PTMs in regulating diverse biological processes, an important future direction is to extract not only modification events but also their functional impacts on biological activity. Furthermore, large-scale running of our framework could facilitate the systematic extraction of PTM information for understudied or minimally reported PTMs, enabling the construction of a comprehensive and continuously updated PTM knowledge resource.

Finally, GenPTM establishes a new direction for PTM-type independent event extraction and provides a scalable tool for automated PTM knowledge discovery in proteomics research and related domains.

## Supplementary Material

Supplementary Files

This is a list of supplementary files associated with this preprint. Click to download.

- GenPTMsupplementary.pdf

## Figures and Tables

**Table 1:** Mapping table of PTM terms to generalized modification and group classes

	Generic Placeholder Class	
PTM Type	MODIFICATION	GROUP
Ubiquitination	Ubiquitination, ubiquitinated, monoubiquitinated, etc.	Ubiquitin, Ub, etc.
Sumoylation	Sumoylation, sumoylated, etc.	SUMO-1, SUMO-2, etc.
Neddylation	Neddylation, neddylated, etc.	NEDD8, etc.
Acetylation	Acetylation, acetylated, acetylating, etc.	Acetyl
Phosphorylation	Phosphorylation, phosphorylated, etc.	Phosphate
Palmitoylation	Palmitoylation, palmitoylated, etc.	Palmitate
ADP-Ribosylation	ADP-ribosylated, ADP-ribosylates, etc.	ADP-ribose
Succinylation	Succinylation, succinylated, etc.	Succinyl
Hydroxylation	Hydroxylation, hydroxylated, etc.	Hydroxyl
UFMylation	UFMylation, UFMylated, UFMylating	UFM1, UFM, etc.
Citrullination	Citrullination, citrullinated, citrullinating, etc.	Citrulline
AMPylation	AMPylates, di-AMPylation, etc.	AMP moiety
Lipoylation	lipoylated, lipoylating, etc.	Lipoyl

**Table 2:** Abstract-level performance of GenPTM across different PTM categories

	<protein, site> pair			protein-only			site-only		
PTM Category	P	R	F1	P	R	F1	P	R	F1
WS-PTMs	98.57%	89.61%	93.88%	91.78%	97.10%	94.37%	96.59%	95.51%	96.05%
OF-PTMs	96.77%	96.77%	96.77%	87.63%	94.77%	91.06%	100.00%	96.88%	98.41%
LF-PTMs	70.37%	82.61%	76.00%	87.50%	92.11%	89.74%	81.08%	100.00%	89.55%
All PTMs	93.08%	91.36%	92.21%	89.66%	95.68%	92.58%	94.65%	96.72%	95.68%

## Data Availability

The training and evaluation datasets were manually annotated in this study using abstracts collected from publicly available sources, including iProLINK (https://research.bioinformatics.udel.edu/iprolink/), BioGRID (https://thebiogrid.org/), PubMed (https://pubmed.ncbi.nlm.nih.gov/) and dbPTM (https://biomics.lab.nycu.edu.tw/dbPTM/). The annotated datasets (both training and evaluation) generated and analysed during this study, together with the source code, are available at https://github.com/udel-biotm-lab/GenPTM.

## Footnotes

1
https://www.ncbi.nlm.nih.gov/home/develop/api/

2
https://spacy.io/

3
https://huggingface.co/transformers/

4
https://sites.google.com/site/bionlpst/bionlp-shared-task-2011

5
https://2013.bionlp-st.org/
